# Psychiatrists’ Insights on Integrating Occupational Therapy in Mental Health Care: A Multisite Middle Eastern Study

**DOI:** 10.3390/ijerph21080974

**Published:** 2024-07-26

**Authors:** Naser Alotaibi, Hamad Alhamad, Haitham Jahrami, Muhammad O. Al-Heizan, Lujane Albaghli, Hasan Ashkanouni, Hashem Abu Tariah, Hamad Abouelhassan, Moh Alkhamis

**Affiliations:** 1 Occupational Therapy Department, Faculty of Allied Health Sciences, Kuwait University, P.O. Box 31470, Sulaibekhat 90805, Kuwait; h.alhamad@ku.edu.kw; 2 Department of Psychiatry, College of Medicine and Medical Sciences, Arabian Gulf University, Manama P.O. Box 26671, Bahrain; haitham.jahrami@outlook.com; 3 Governmental Hospitals, Manama P.O. Box 12, Bahrain; 4 Department of Health Rehabilitation Sciences, College of Applied Medical Sciences, King Saud University, Riyadh 12372, Saudi Arabia; malheizan@ksu.edu.sa; 5 Occupational Therapy Department, Kuwait Center for Mental Health, Ministry of Health, Kuwait City 12009, Kuwait; lujaneot@gmail.com (L.A.); hassanash-1994@hotmail.com (H.A.); 6 Department of Occupational Therapy, Faculty of Applied Medical Sciences, Hashemite University, Zarqa 13133, Jordan; hashem@hu.edu.jo; 7 Department of Epidemiology and Biostatistics, Faculty of Public Health, Kuwait University, Kuwait City 13110, Kuwait; hamad.abouelhassan@hscph.ku.edu.kw (H.A.); m.alkhamis@ku.edu.kw (M.A.)

**Keywords:** interprofessional collaboration, referral, occupational therapy autonomy, Middle East, rehabilitation, mental health, psychiatry, multidisciplinary

## Abstract

**Objective**: The purpose of this study was to explore the knowledge, perception, attitude, and self-efficacy of psychiatrists regarding the role of occupational therapy in mental health practice. **Materials and Methods**: This study utilized a cross-sectional design to examine the perspectives of psychiatrists from various Middle Eastern countries on occupational therapy practice. A self-developed tool was mainly used to assess occupational therapy knowledge, autonomy, attitude, and self-efficacy. STATA version 16.0 was employed for all subsequent statistical analyses. The data were analyzed using the Kruskal–Wallis and chi-square tests. **Results**: A total of 117 participants (psychiatrists) from various Middle Eastern countries, including Kuwait, Bahrain, Saudi Arabia, and Jordan, took part in this study. Overall, no significant differences were found between the sites in terms of knowledge and self-perception of occupational therapy autonomy, indicating a lack of understanding about the unique nature of occupational therapy as a distinct health care profession (*p*-values > 0.05). However, this study’s participants demonstrated a positive attitude and self-efficacy towards occupational therapy. **Conclusions**: In order to promote the desired therapeutic outcomes, a referral form for occupational therapy, encompassing the main areas of intervention, was proposed. Such a referral form can help inform psychiatrists about the key components of occupational therapy services in mental health practice, thus facilitating the desired interprofessional collaboration and patient outcomes. This study’s implications and future directions are also discussed.

## 1. Introduction

Collaboration and communication between healthcare professionals are pivotal for the comprehensive treatment of patients with mental health issues [1,2]. Among these professionals, psychiatrists and occupational therapists play crucial roles, each bringing unique perspectives and skills to patient care [2,3]. Psychiatrists, with their expertise in diagnosing mental health conditions and prescribing medication, along with occupational therapists, who focus on improving patients’ functional abilities through everyday activities, both aim to enhance the quality of life for those with mental health conditions [2,3,4]. However, the extent to which psychiatrists recognize and value the role of occupational therapists can greatly affect the effectiveness of collaborative practices and, ultimately, patient outcomes. This mutual understanding is imperative for fostering a unified approach to mental health care and promoting patients’ physical and psychological well-being [4,5]. Interdisciplinary collaboration in mental health care is the gold standard for delivering patient-centered care through the cohesive efforts of diverse professionals [6,7]. The synergy between psychiatrists and occupational therapists is particularly significant, given their complementary roles in addressing both the psychological and practical aspects of mental health conditions [5,8]. Effective collaboration enhances patient outcomes and promotes a more holistic approach to mental health care, ensuring that patients receive comprehensive support, which spans medical treatment and functional rehabilitation [7].

A holistic approach in healthcare and research refers to considering the whole system rather than just individual parts. This means looking at the interconnectedness of various factors and addressing a wide range of influences, including physical, mental, emotional, social, and environmental aspects. In the context of our study, a holistic approach would involve understanding not only the specific knowledge healthcare professionals have about occupational therapy but also how this knowledge integrates with their overall practice, teamwork dynamics, and patient care outcomes. Therefore, it is crucial to understand psychiatrists’ perceptions of the roles and contributions of occupational therapy in mental health practice.

The perception of occupational therapy by psychiatrists can vary widely and is often influenced by their exposure to and understanding of the field [9]. While some psychiatrists recognize the valuable contributions of occupational therapists in developing comprehensive treatment plans, others may have a limited view of their role, primarily associating it with basic activities of daily living [10]. This discrepancy can be attributed to several factors, including the level of interdisciplinary interaction during psychiatric training, experiences with collaborative practices, and the prevailing culture within healthcare institutions regarding teamwork [7,9]. Misconceptions about the scope of occupational therapy can lead to its underutilization in mental health settings, potentially compromising the quality of patient care.

Occupational therapy plays a crucial role in psychiatric rehabilitation, as highlighted in various research papers [11,12,13,14,15,16]. These studies emphasize the significance of occupational therapy in psychiatric institutions, showcasing its evolution over time and its impact on patient care and well-being. Additionally, the role of occupational therapists in the acute psychiatric setting is explored, focusing on assessment, intervention, and discharge planning, which are essential components of psychiatric training [17,18]. Furthermore, occupational therapists aim to enable activity engagement by enhancing people’s abilities and opportunities or by modifying their environments. They offer a wide range of interventions that address cognitive, emotional, and social needs, facilitating patients’ reintegration into society and improving their independence and quality of life [19,20]. However, the lack of awareness among psychiatrists regarding these contributions can hinder the integration of occupational therapy into mental health care plans, thereby limiting its therapeutic services and patient care [9,21].

Occupational therapy practice is relatively new in the Middle East, but the associated services may not be fully understood [2]. Additionally, it is crucial to enhance psychiatrists’ understanding of OT in order to foster effective interdisciplinary collaboration [9]. However, to our knowledge, there are no existing research studies exploring the knowledge, perception, attitude, and self-efficacy of psychiatrists regarding the role of OT in mental health practice, particularly in the Middle Eastern context. Therefore, the present study aimed to explore the knowledge, perception, attitude, and self-efficacy of psychiatrists concerning the role of OT in mental health practice in the Middle East. To fulfill this gap in the literature, information was obtained from psychiatrists in different Middle Eastern countries, including Kuwait, Saudi Arabia, Bahrain, and Jordan. Notably, Kuwait, Saudi Arabia, and Jordan have well-established occupational therapy educational programs accredited by the World Federation of Occupational Therapists [22].

## 2. Materials and Methods

### 2.1. Design

This study utilized a descriptive and cross-sectional research design.

### 2.2. Participants and Settings

The sample included psychiatrists who worked in governmental hospitals. The inclusion criteria were as follows: (1) psychiatrists working in governmental hospitals; (2) psychiatrists treating adults, children, or both; and (3) psychiatrists from Middle Eastern countries where occupational therapy services are offered in these governmental hospitals. The exclusion criteria were as follows: (1) psychiatrists working only in private practices; and (2) psychiatrists working in facilities that do not provide occupational therapy services. We excluded private hospitals or those without OT services to ensure that participants had relevant experience with OT in their practice settings. This decision was based on our belief that the availability and integration of OT services can vary significantly between public and private healthcare settings. In other words, psychiatrists in private practice may have different experiences and less interaction with occupational therapists, which could introduce variability that does not align with this study’s objectives. By excluding them, we aimed to create a more homogenous sample that better represents psychiatrists’ knowledge and perspectives within institutional environments.

### 2.3. Instrumentation

In order to address this study’s purpose, experts in the field of occupational therapy designed a self-developed questionnaire. The expert review process was a critical component in refining and validating our questionnaire. Five occupational therapists, each with over 5 years of specialized experience in mental health, were carefully selected for their diverse expertise and recognized contributions to the field. These experts independently reviewed the initial questionnaire, providing comprehensive feedback on the content validity, clarity, relevance, and cultural sensitivity of each item. Their suggestions ranged from minor wording adjustments to recommendations for additional questions and structural changes. To illustrate, additional intervention components reflecting occupational therapy contributions were added including, for example, sensory integration, cognitive retraining, behavioral intervention, social skills training, and academic skills training. The inclusion of comprehensive intervention components can guide occupational therapy intervention and ensure the provision of holistic patient care. In addition, it will facilitate the desired interprofessional communication between psychiatrists and occupational therapists in psychiatric rehabilitation.

Following this initial review, this revised version underwent a second round of expert review to ensure that all modifications adequately addressed the initial feedback. In cases where expert opinions diverged significantly, we facilitated a consensus-building process through direct communication or mediated discussions. This iterative approach, culminating in final approval from all experts, ensured that our questionnaire benefited from a comprehensive range of professional insights, thereby enhancing its validity and practical relevance in the field of occupational therapy and mental health. In other words, the expert panel reached a consensus on the questionnaire’s relevance and appropriateness, ensuring that it reflected the intended purpose. The questionnaire consisted of a variety of questions, organized into six components.

#### 2.3.1. Demographic Data

This component contains 11 items, including age, gender, educational level, country of practice, years of working experience, common diagnoses encountered, awareness of the occupational therapy profession, years of working experience with occupational therapists, attendance of workshops/seminars related to occupational therapy, frequency of attendance, and number of referrals to occupational therapy services.

#### 2.3.2. Self-Perception of Occupational Therapy (OT) Autonomy

This component contains 3 items: (1) acknowledgment of the distinct role of occupational therapy in mental health practice, (2) recognition of key differences between the occupational therapist and the psychologist, and (3) recognition of key differences between the occupational therapist and the social worker. Understanding the OT identity and respecting its values and contributions, particularly in the area of mental health practice, is an important focus in OT practice. In other words, maintaining OT autonomy is essential and facilitates desired interprofessional collaboration, promoting a healthier clinical environment [14,23,24,25]. Notably, the main rationale for including psychologists and social workers in this section is that it is common in mental health settings for misconceptions and lack of role clarity to exist among psychiatrists regarding the specific responsibilities and contributions of occupational therapists, psychologists, and social workers [9]. In addition, similarities could exist between these health care professions as the main ones within mental health settings, possibly leading to role ambiguity [12].

#### 2.3.3. Knowledge about Major Components of Occupational Therapy (OT) Intervention Focus

This component includes 24 items, from which participants are asked to identify only the items that clearly reflect the intervention focus of occupational therapy in mental health practice. Items reflecting the occupational therapy intervention focus include activities of daily living, recreational leisure activities, sensory integration group, lifestyle and daily routines, behavioral intervention, substance use group, cognitive training, anxiety/stress management, academic skills training, assertiveness training, pre/vocational training, coping strategies, social skills training group, and rest and sleep. Since there could be overlaps or similarities in the intervention focus among health care disciplines in mental health practice, having adequate knowledge about the major components of the OT intervention focus contributes to minimizing role confusion and ambiguity, and therefore supports the desired referral services and the provision of holistic service delivery [2,11,12].

#### 2.3.4. Attitude towards Occupational Therapy (OT)

This component contains three items that mainly reflect the attitude towards occupational therapy as a health care profession. These items include (1) acknowledging the importance of the occupational therapy profession as an equally vital profession in the field of mental health practice, (2) recognizing the valuable contributions of occupational therapy in mental health practice, and (3) acknowledging the agreement of psychiatrists in referring patients with mental health conditions to receive occupational therapy services. Understanding the attitudes of psychiatrists concerning occupational therapy in mental health can be an important factor in fostering positive collaboration and respect within various health care environments [2].

#### 2.3.5. Self-Efficacy Regarding Occupational Therapy (OT)

(1) Self-efficacy in mental health practice entails the level of motivation to work with occupational therapists in the field of mental health practice and (2) confidence in the skills and contributions of occupational therapists in evaluating and treating patients with mental health conditions. The concept of self-efficacy in mental health practice can play a crucial role in promoting positive relationships and support among health care professionals and patients. Therefore, we believe that assessing the self-efficacy regarding occupational therapy, from the perspective of psychiatrists, is an important indicator of collaboration in practice [18].

#### 2.3.6. Satisfaction with One’s Own Knowledge Base of Occupational Therapy (OT) Contributions to Patient Rehabilitation

This component contains one item relevant to satisfaction with one’s own knowledge base regarding occupational therapy contributions to mental health practice. It assesses the psychiatrist’s satisfaction level with their knowledge base concerning OT in mental health practice, thus providing a better understanding of its relationship with the actual knowledge acquired. This approach can assist in better understanding their perception and whether an increased level of awareness should be emphasized accordingly [26].

For the reader’s convenience and better understanding of the questionnaire’s items and responses, the entire questionnaire is available in Supplementary File S1.

### 2.4. Procedure

Ethical approval was obtained from the Institutional Review Board of Kuwait University. This study adhered to data protection laws, specifically the General Data Protection Regulation (GDPR) and local regulations. This study was conducted in two stages. The first stage was a pilot study performed by 10 psychiatrists working in mental health hospitals. The pilot test was successfully performed to gain insight into the relevance and appropriateness of the questionnaire and to assess the participants’ comprehension of the questions. During discussions with the participants after the questionnaire administration, the instrument was found to be clear and relevant. No floor or ceiling effects were found, thus supporting its content validity. The second stage was the main study. All psychiatrists were contacted in person by occupational therapists in their facility and/or online using available social media platforms.

### 2.5. Data Analysis

We used STATA version 16.0 to conduct all the subsequent statistical analyses. We estimated the median and interquartile (IQ) range of the quantitative variables while summarizing the categorical variables into frequencies and relative frequencies. We investigated statistically significant differences and relationships in the responses among sites (i.e., countries) using Kruskal–Wallis and chi-square Fisher’s exact tests, respectively. We set the significance level for the statistical tests at an α = 0.05. Tables and figures were then generated to best represent the results.

## 3. Results

The total sample included 117 participants (psychiatrists) representing different Middle Eastern countries, including Bahrain, Kuwait, Saudi Arabia, and Jordan. The participants’ median age was 40 years, and their IQ ranged between 33 and 50. Most participants (*n* = 49, 41%) were from the Kingdom of Bahrain, while the fewest were from Jordan (*n* = 4, 3.4%). Twenty-six percent of the participants had between 5 and 10 years of clinical experience. Schizophrenia was the most commonly diagnosed mental illness (26%), followed by major depressive disorder (21.4%), bipolar affective disorder (15.4%), addiction (15.4%), and dementia (13.7%). Thirty-four percent of the participants had more than 10 years of experience dealing with OTs. Moreover, 33% of the respondents referred their clients to OT services at least 6 to 10 times (Table 1).

**Figure 1:**
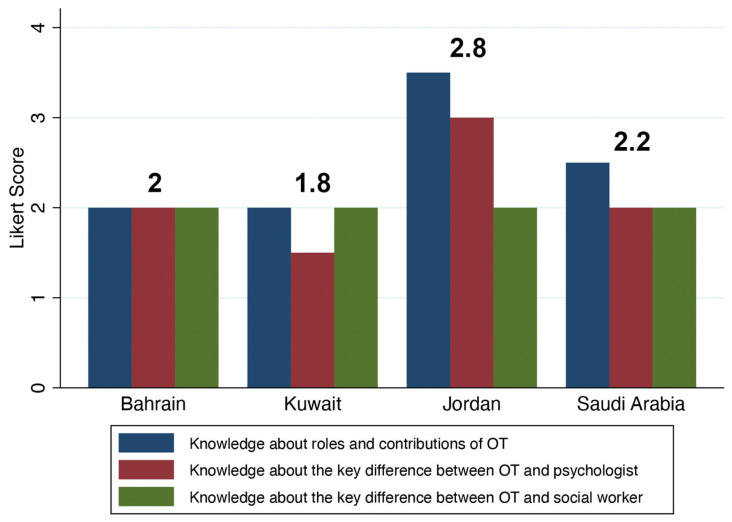
Bar chart showing the median differences in self-perception of occupational therapy (OT) autonomy between sites using the Likert scale. The numbers on the top of the bar chart indicate the value of the overall median score for each site. The Likert scale on the Y-axis comprised strongly disagree = 0, disagree = 1, not sure = 2, agree = 3, and strongly agree = 4.

We did not find any significant differences between sites in terms of self-perception of occupational therapy autonomy. This generally indicates a lack of understanding about the unique nature of occupational therapy as a distinct health care profession (*p*-values > 0.1; Table 2 and Figure 1), with an overall median score of 2. Additionally, the overall median score for each site, except for Jordan, was approximately 2 (Figure 1). On the other hand, the participants in this study exhibited a generally positive attitude and self-efficacy towards occupational therapy. They also expressed moderate satisfaction with their knowledge base regarding the contributions of occupational therapy in mental health practice. Therefore, there were no significant relationships between the sites and attitude, self-efficacy, or knowledge of occupational therapy (*p*-values > 0.5; Table 3).

As reported in Table 4, there were contrasting perspectives regarding the participants’ knowledge about the main components of occupational therapy intervention focus. For example, the majority of this study’s participants correctly identified rest and sleep, activities of daily living, recreation and leisure activities, lifestyle and daily routines, substance use groups, and cognitive retraining as the main areas of practice relevant to occupational therapy. Conversely, more than half of this study’s participants incorrectly identified speech articulation skills, medication administration, gait training, and medical equipment setup as the main components of occupational therapy intervention focus. Moreover, except for the relationship between sites and solving family problems as a major component of OT intervention (*p* = 0.04), all the other components had no significant relationships with the participants’ sites (*p*-values > 0.1; Table 4).

## 4. Discussion

This study aimed to explore the knowledge base, perception, attitude, and self-efficacy of psychiatrists regarding the role of occupational therapy in mental health practice. It was a multisite study investigating perspectives in the countries of Kuwait, Bahrain, Jordan, and Saudi Arabia. The most common clinical diagnoses were schizophrenia, depression, bipolar affective disorder, anxiety disorders, and addiction. Therefore, an in-depth understanding of the clinical features, manifestations, and functional deficits of such disorders should be emphasized within occupational therapy educational curricula and should be addressed by occupational therapists in the clinical environment. Hence, as core concepts and fundamental beliefs in occupational therapy theory and practice, occupational therapists support the engagement of patients with mental health conditions in healthy patterns of activity and promote meaningful participation in communities targeting patients’ recovery, health, and wellbeing. There is a growing body of evidence that supports the uniqueness and contribution of occupational therapists in the treatment and management of patients who present with various mental health diagnoses, including adults and children [15,20,27,28,29,30,31]. As indicated in this study, the participants worked with occupational therapists, attended workshops relevant to occupational therapy, and referred patients to receive occupational therapy services. However, approximately half of the psychiatrists did not recognize the key contributions of occupational therapy in mental health practice and were less knowledgeable about the key differences between occupational therapists and other health care professionals, such as psychologists and social workers. This could be due to ambiguity about the specific role of occupational therapy in mental health practice and limited understanding of its uniqueness. As a result, role confusion among various health care professionals occurs, thus resulting in fragmented services and diminished therapeutic care [2]. Additionally, this could mitigate the need for the desired referral services for various patient populations [32]. Nevertheless, occupational therapists are encouraged to apply occupation-based interventions, promote their professional identity, and engage in recovery-oriented and collaborative practice, thus facilitating role recognition, increased referral, and desired patient service provision [32,33].

Furthermore, we should closely consider the impact of the lack of knowledge concerning the areas of practice in which occupational therapists are mainly involved during the provision of their therapeutic services. For example, more than half of this study’s participants considered speech articulation skills, medication administration, gait training, and medical equipment setup to be the main components of occupational therapy intervention focus. This could contribute to limited recognition of occupational therapists’ role identity, as well as their professional values and goals, leading to diminished rehabilitation services [2,34,35,36].

In contrast, the present study provides evidence that the majority of this study’s participants are oriented and knowledgeable about the main components of occupational therapy intervention focus, including rest and sleep, activities of daily living, recreation and leisure activities, lifestyle and daily routines, substance use groups, and cognitive retraining; this finding is also supported by the literature [35,36,37,38,39,40,41]. The results of this study also indicate that this study’s participants exhibited a positive attitude and self-efficacy regarding the occupational therapy services provided within mental health practice. Hence, this approach is encouraging and serves the interdisciplinary collaboration process, thus promoting more integrated therapeutic outcomes [2,42]. Therefore, the general positive attitude and self-efficacy of this study’s participants concerning occupational therapy contributions is an indication of the recognition of the key role of occupational therapists in the management of patients with mental health conditions; this could also be attributed to the positive therapeutic outcomes reported by the patients, as well as the observed functional performance of patients receiving occupational therapy services [2].

In addition, the results revealed a lack of full understanding of the role of occupational therapists in mental health settings. Such confusion about role overlap between occupational therapists and other health care professionals is still prevalent. Thus, the interprofessional nature of practice in mental health settings may have resulted in a blurred understanding by psychiatrists of the distinct role of occupational therapists. These findings are consistent with those of previous studies [36]. Furthermore, these gaps in knowledge indicate a need for more targeted, clear advocacy of the distinct role of OTs in mental health. This could be achieved through educational programs specifically targeting psychiatrists or incorporating clinical rotations or internships in occupational therapy within psychiatric residency programs. Consequently, this can provide psychiatrists with direct exposure to occupational therapy practices [43].

Although the psychiatrists generally reported a positive attitude towards the value of occupational therapy services in mental health practice, approximately one-third of them expressed uncertainty about the value of such services and whether they would refer patients to occupational therapy. This uncertainty can be attributed to challenges in aligning professional values with the capacity to incorporate an occupational perspective in settings that are predominantly influenced by a biomedical model, as is often the case in psychiatric settings [35]. Additionally, there is a need for a clearer definition and understanding of collaboration within the field of occupational therapy [44]. It is also suggested that increased involvement in collaborative research projects by both psychiatrists and occupational therapists could provide firsthand experience of the benefits of occupational therapy [45]. Occupational therapy educational programs could also play a vital role in emphasizing occupation-centered practice within educational settings, assisting future occupational therapists working in mental health settings to effectively demonstrate the unique value of occupational therapy [46]. Moreover, the psychiatrists’ satisfaction level with their knowledge base of occupational therapy contributions in mental health practice was moderate, further highlighting the need for additional education on the intervention focus areas of occupational therapy practice. Occupational therapists also bear a greater responsibility to advocate for their pivotal role as an autonomous health care profession that can significantly contribute to the health and well-being of patients within interdisciplinary rehabilitation teams [26].

Given this study’s findings and to address concerns regarding role ambiguity and lack of clarification regarding the autonomy of occupational therapy in mental health practice in various Middle Eastern countries, we highly recommend developing a well-structured and comprehensive referral form. This form should reflect the unique nature of occupational therapy services for evaluating and treating patients with various mental health conditions. For the convenience of readers, this form is included in Supplementary File S2. The referral form serves multiple purposes. Firstly, it acts as the main communication tool between occupational therapists and psychiatrists, facilitating the understanding of the key domains and areas targeted by occupational therapists during the patients’ therapeutic process. Secondly, it promotes interprofessional collaboration, ensuring a healthy and productive clinical atmosphere. Thirdly, it minimizes role ambiguity and service duplication, supporting comprehensive and integrated service provision. Most importantly, implementing this systematic referral process will foster trust among rehabilitation team members and ultimately contribute to positive and fruitful patient outcomes.

The current study has important implications worth mentioning. Firstly, this study is the first of its kind in the Middle East to investigate the knowledge base, perception, attitude, and self-efficacy of psychiatrists regarding occupational therapy services in mental health practice. This study includes samples from various Middle Eastern countries where occupational therapy services are relatively developed, such as Kuwait, Saudi Arabia, Bahrain, and Jordan. This multisite study will encourage further collaborative efforts in the region pertaining to mental health practice, which is a significant aspect of the occupational therapy profession. Of note, the approximate number of occupational therapists in relation to 100,000 inhabitants for the states of Kuwait, Saudi Arabia, Bahrain, and Jordan, is around 250 (6%), 1150 (3%), 16 (1%), and 1900 (17%), respectively. However, such statistics are an estimation and are based on subjective information, since there is a lack of published occupational therapy literature regarding detailed and up-to-date statistics. Thus, to ensure sufficient supply of occupational therapists in these countries, future studies are warranted to tackle issues pertained to national and regional workforce research to meet competency standards in occupational therapy practice. In turn, the incorporation of cross-national research collaboration in this area can advance occupational therapy services and subsequently address the population health and occupational needs [46].

The Middle East presents a unique socio-cultural and healthcare landscape that significantly influences the practice and perception of occupational therapy in mental health settings. Cultural sensitivity is crucial in this region, where deeply ingrained beliefs and values profoundly impact mental health treatment approaches and the acceptance of interdisciplinary practices [47,48]. The cultural context shapes not only patient attitudes but also healthcare professionals’ perspectives, potentially affecting their openness to collaborative care models [47,48]. The healthcare infrastructure in Middle Eastern countries often differs from that of Western nations, with varying levels of resources and structural organizations that can impact the implementation and collaboration between different health disciplines. Furthermore, the educational background of healthcare professionals, including psychiatrists and occupational therapists, may diverge from international norms, leading to disparate levels of awareness and understanding of each other’s roles within the mental health care team [47,48].

Given the unique regional characteristics of the Middle East/Arab countries, research conducted in other parts of the world may not fully capture or address the specific challenges and opportunities present in this region. This highlights the importance of conducting studies that are specific to the Middle East in order to ensure that the findings are relevant, applicable, and can effectively address the local challenges in integrating occupational therapy within mental health practice. By focusing research efforts within the Middle East, we can gain insights into the dynamics of interprofessional collaboration in this cultural context, understand the barriers to the recognition and utilization of occupational therapy in mental health settings, and develop customized strategies for enhancing collaborative care. Such regionally focused research is crucial for informing policy decisions and shaping educational curricula. Therefore, while considering the cultural contexts, we firstly highly encourage the value of interprofessional education within the health science curricula to ensure the understanding of each profession’s roles and contributions in mental health practice, leading to desired interprofessional collaboration in the future clinical environments [49].

Secondly, the findings of this study can provide insight into the referral system as an approach that aids occupational therapists in clarifying their role and contributions to the rehabilitation team. This will help mitigate the limited understanding of the holistic service provision for patients with mental health conditions. However, it is important to carefully examine the proposed referral form and its applicability within mental health practice. Emphasis should be placed on clarifying the main domains and elements of the form for the rehabilitation team members. Furthermore, occupational therapists should take the initiative to spread awareness of the form to psychiatrists and explain its rationale and benefits. This could be achieved through educational workshops, where psychiatrists can be informed about the scientific evidence and efficacy of the specific elements of the referral form during the rehabilitation process.

Thirdly, it is crucial for occupational therapists working in mental health settings to utilize their own clinical reasoning skills, as well as base their practices on occupational therapy theoretical frameworks and practice models. This will promote the identity of occupational therapy and support its uniqueness, as a distinct health care profession in mental health practice [25]. Fourthly, in the context of multidisciplinary approaches utilized in practice, it is important to document and measure the effectiveness of occupational therapy intervention services within the naturalistic context of psychiatric rehabilitation. To illustrate, occupational therapists are highly encouraged to publish research studies involving rigorous designs, thus providing solid evidence for the key role of occupational therapy in mental health practice [16]. This is particularly important in countries where occupational therapy is relatively new. By following this approach, the value of occupational therapy can be demonstrated, and the effectiveness of its services can be assessed using cross-culturally adapted tools.

Lastly, educational programs in these countries, including Kuwait, Saudi Arabia, and Jordan, are approved by the World Federation of Occupational Therapists. Hence, it is highly encouraged for faculty members to encourage their students to be strong advocates of their profession after graduation. For example, the graduates of these programs should be geared toward emphasizing the value of interprofessional collaboration during the therapeutic process and finding ways and strategies to raise awareness about occupational therapy among the rehabilitation team. In this regard, we also encourage occupational therapy associations and governing bodies to play an active role in legislating laws and regulations that support their professional autonomy, specify their job descriptions, and clearly inform their intervention focus areas and contributions within various practice areas, including mental health practice.

### Limitations

The current study has several limitations that deserve attention. Firstly, the samples chosen were not equally representative or randomly selected, limiting the generalizability of this study’s findings. However, this study’s samples of the Kuwaiti and Bahraini psychiatrists represented 41.9% and 77.8% of the total population samples, respectively. These samples are therefore adequately informative and provide a clear picture of their insight, knowledge, and level of understanding concerning occupational therapy in mental health practice. Secondly, the validation of the developed questionnaire was not fully addressed. Therefore, further exploration of the psychometric properties of the developed tool is warranted. Thirdly, the rationale of the psychiatrists’ knowledge and perception about the uniqueness and contributions of occupational therapists in mental health practice was not fully explored. Therefore, future qualitative studies are encouraged to identify the facilitators and barriers influencing their understanding of occupational therapy knowledge and contributions. This approach can provide an in-depth understanding of their perspectives as well as illuminate the underlying reasons for their misconceptions of the occupational therapy role in mental health practice, thereby promoting the desired interprofessional collaboration and facilitating optimal service delivery. Lastly, this study’s findings were not conclusive for all Middle Eastern countries in which occupational therapy services are offered. Therefore, future studies are needed to capture the perspectives of psychiatrists in other countries, thus possibly providing a deeper understanding and further insights regarding their knowledge and perceptions about the role of occupational therapy in mental health practice.

## 5. Conclusions

The results of this study indicate that the knowledge base of occupational therapy, as well as the self-perception of occupational therapy autonomy in mental health practice among psychiatrists in Middle Eastern countries, are not fully understood. However, their attitude and self-efficacy related to occupational therapy practice were generally positive. Therefore, occupational therapists working in the clinical environment are more likely to support interprofessional collaboration, with psychiatrists serving as the main source of patient referrals for receiving occupational therapy services. To promote desired therapeutic outcomes, a proposed occupational therapy referral form highlights the main occupational therapy domains and components relevant to areas of intervention. This form can inform psychiatrists about the key components and focuses of occupational therapy services in mental health practice, support interprofessional communication, and ultimately lead to the desired patient services. It is the responsibility of occupational therapists in mental health practice to play an active role and be strong advocates for their roles and contributions in this area of practice. For example, they can provide educational workshops or seminars for psychiatrists regarding all the components of the referral form, highlighting the intervention focus and therapeutic value of each component, as supported by evidence.

Moreover, this study highlights the critical need for improved understanding and collaboration between psychiatrists and occupational therapists. By addressing misconceptions and promoting educational initiatives, the mental health care system can move toward a more integrated model that fully embraces the contributions of occupational therapy. This shift is essential for delivering holistic, patient-centered care that effectively addresses the wide range of needs encountered in mental health practice. Therefore, we suggest the development of regional initiatives among occupational therapists in different Middle Eastern countries, focusing on strategies to promote occupational therapy values and contributions to all rehabilitation team members in the area of mental health practice. Finally, the emphasis on interprofessional education within the health science curricula is a key factor in facilitating future interprofessional collaboration in psychiatric rehabilitation, thus reflecting effective patient service delivery.

## Figures and Tables

**Table 1:** Baseline characteristics of the participants (*n* = 117).

Characteristics	Mean [IQ ^†^] or n (%)
**Age**	40 [33, 50]
**Gender**	
Male	64 (54.7%)
Female	53 (45.3%)
**Site**	
Bahrain	49 (41.9%)
Kuwait	36 (30.8%)
Jordan	4 (3.4%)
Saudi Arabia	28 (23.9%)
**Clinical Experience**	
less than a year	2 (1.7%)
1–5 years	25 (21.4%)
5–10 years	31 (26.5%)
11–15 years	5 (4.3%)
16–20 years	24 (20.5%)
21–25 years	19 (16.2%)
30+ years	11 (9.4%)
**Most Common Diagnoses**	
Addiction	18 (15.4%)
Anxiety	6 (5.1%)
* ASD	3 (2.6%)
* BAD	18 (15.4%)
Dementia	16 (13.7%)
* MDD	25 (21.4%)
Schizophrenia	31 (26.5%)
**Experience with OT**	
No experience	17 (14.5%)
Less than one year	9 (7.7%)
1–5 years	31 (26.5%)
6–10 years	20 (17.1%)
More than 10 years	40 (34.2%)
**Attending OT Workshop**	
Yes	70 (59.8%)
No	47 (40.2%)
**Number of Workshops**	
Once	32 (27.3%)
Twice	17 (14.5%)
Three times	50 (42.7%)
More than three times	18 (15.4%)
**Number of Referrals to OT**	
None	2 (1.7%)
1 to 5	41 (35%)
6 to 10	39 (33.3%)
>10	35 (29.9%)

^†^ Interquartile range; * ASD = autism spectrum disorder; BAD = bipolar affective disorder; MDD = major depressive disorder.

**Table 2:** Differences in self-perception of occupational therapy autonomy between sites. The Likert scale comprised strongly disagree = 0, disagree = 1, not sure = 2, agree = 3, and strongly agree = 4.

	Median (IQ ^a^)	*p*-Value ^b^
Knowledge about roles and contributions of OT	2 (1–3)	0.18
Knowledge about the key difference between occupational therapist and psychologist	2 (1–3)	0.47
Knowledge about the key difference between occupational therapist and social worker	2 (1–3)	0.82

^a^ Interquartile range; ^b^ *p*-values obtained by the Kruskal–Wallis test.

**Table 3:** Attitude, self-efficacy, and knowledge satisfaction toward occupational therapy between sites. The options “disagree” and “strongly disagree” were not selected by any of the participants.

	*n* (%)			*p*-Value *
	Neutral	Agree	Strongly Agree	
**Attitude**				
*I believe occupational therapy is an equally important health care profession as other health care professions related to the rehabilitation of patients in mental health practice.*				0.73
Bahrain	25 (51.0%)	19 (38.8%)	5 (10.2%)	
Kuwait	18 (50.0%)	14 (38.9%)	4 (11.1%)	
Jordan	1 (25.0%)	3 (75.0%)	0	
Saudi Arabia	16 (57.1%)	8 (28.6%)	4 (14.3%)	
*I value the role of occupational therapy in the rehabilitation of patients in mental health practice.*				0.76
Bahrain	15 (30.6%)	24 (49.0%)	10 (20.4%)	
Kuwait	13 (36.1%)	17 (47.2%)	6 (16.7%)	
Jordan	1 (25.0%)	1 (25.0%)	2 (50.0%)	
Saudi Arabia	9 (32.1%)	15 (53.6%)	4 (14.3%)	
*I would refer patients to receive occupational therapy services in mental health practice.*				0.92
Bahrain	15 (30.6%)	18 (36.7%)	16 (32.7%)	
Kuwait	10 (27.8%)	13 (36.1%)	13 (36.1%)	
Jordan	1 (25.0%)	2 (50.0%)	1 (25.0%)	
Saudi Arabia	10 (35.7%)	12 (42.9%)	6 (21.4%)	
**Self-efficacy**				
*I am motivated to work with occupational therapists as part of the rehabilitation team.*				0.99
Bahrain	18 (36.7%)	22 (44.9%)	9 (18.4%)	
Kuwait	15 (41.7%)	15 (41.7%)	6 (16.7%)	
Jordan	1 (25.0%)	2 (50.0%)	1 (25.0%)	
Saudi Arabia	9 (32.1%)	14 (50.0%)	5 (17.9%)	
*I am confident in the skills and contributions of occupational therapist for evaluating and treating patients with mental illness.*				0.56
Bahrain	20 (40.8%)	20 (40.8%)	9 (18.4%)	
Kuwait	12 (33.3%)	16 (44.4%)	8 (22.2%)	
Jordan	2 (50.0%)	2 (50.0%)	0	
Saudi Arabia	16 (57.1%)	9 (32.1%)	3 (10.7%)	
**Satisfaction**				
*I am satisfied with my knowledge regarding occupational therapy contributions in mental health practice.*				0.98
Bahrain	20 (40.8%)	17 (34.7%)	12 (24.5%)	
Kuwait	16 (44.4%)	13 (36.1%)	7 (19.4%)	
Jordan	2 (50.0%)	1 (25.0%)	1 (25.0%)	
Saudi Arabia	12 (42.9%)	8 (28.6%)	8 (28.6%)	

* *p*-value obtained by chi-square test.

**Table 4:** Knowledge about major components of occupational therapy (OT) intervention focus.

Intervention Focus	Overall Response	Country				*p*-Value
		Bahrain	Kuwait	Jordan	Saudi Arabia	
**Activities of Daily Living (ADL)**						0.99
Yes	60 (51.3%)	26 (53.1%)	18 (50.0%)	2 (50.0%)	14 (50.0%)	
No	57 (48.7%)	23 (46.9%)	18 (50.0%)	2 (50.0%)	14 (50.0%)	
**Gait Training**						0.59
Yes	63 (53.8%)	26 (53.1%)	17 (47.2%)	3 (75.0%)	17 (60.7%)	
No	54 (46.2%)	23 (46.9%)	19 (52.8%)	1 (25.0%)	11 (39.3%)	
**Counseling**						0.60
Yes	64 (54.7%)	27 (55.1%)	17 (47.2%)	3 (75.0%)	17 (60.7%)	
No	53 (45.3%)	22 (44.9%)	19 (52.8%)	1 (25.0%)	11 (39.3%)	
**Academic Skills Training**						0.80
Yes	58 (49.6%)	24 (49.0%)	16 (44.4%)	2 (50.0%)	16 (57.1%)	
No	59 (50.4%)	25 (51.0%)	20 (55.6%)	2 (50.0%)	12 (42.9%)	
**Recreation/Leisure**						0.17
Yes	63 (53.8%)	27 (55.1%)	21 (58.3%)	0	15 (53.6%)	
No	54 (46.2%)	22 (44.9%)	15 (41.7%)	4 (100.0%)	13 (46.4%)	
**Physical Exercise**						0.74
Yes	59 (49.6%)	24 (49.0%)	19 (52.8%)	1 (25.0%)	15 (53.6%)	
No	58 (50.4%)	25 (51.0%)	17 (47.2%)	3 (75.0%)	13 (46.4%)	
**Solving Family Problems**						0.04 *
Yes	53 (45.3%)	24 (49.0%)	17 (47.2%)	4 (100.0%)	8 (28.6%)	
No	64 (54.7%)	25 (51.0%)	19 (52.8%)	0 (0.0%)	20 (71.4%)	
**Assertiveness Training**						0.99
Yes	57 (48.7%)	24 (49.0%)	18 (50.0%)	2 (50.0%)	13 (46.4%)	
No	60 (51.3%)	25 (51.0%)	18 (50.0%)	2 (50.0%)	15 (53.6%)	
**Sensory Integration Group**						0.79
Yes	54 (46.2%)	23 (46.9%)	18 (50.0%)	1 (25.0%)	12 (42.9%)	
No	63 (53.8%)	26 (53.1%)	18 (50.0%)	3 (75.0%)	16 (57.1%)	
**Helping Patients Find Jobs**						0.86
Yes	54 (46.2%)	23 (46.9%)	17 (47.2%)	1 (25.0%)	13 (46.4%)	
No	63 (53.8%)	26 (53.1%)	19 (52.8%)	3 (75.0%)	15 (53.6%)	
**Psychological Assessments**						0.79
Yes	53 (45.3%)	22 (44.9%)	18 (50.0%)	1 (25.0%)	12 (42.9%)	
No	64 (54.7%)	27 (55.1%)	18 (50.0%)	3 (75.0%)	16 (57.1%)	
**Pre/Vocational Training**						0.99
Yes	53 (45.3%)	22 (44.9%)	16 (44.4%)	2 (50.0%)	13 (46.4%)	
No	64 (54.7%)	27 (55.1%)	20 (55.6%)	2 (50.0%)	15 (53.6%)	
**Thermotherapy**						0.97
Yes	50 (42.7%)	21 (42.9%)	16 (44.4%)	2 (50.0%)	11 (39.3%)	
No	67 (57.3%)	27 (55.1%)	20 (55.6%)	2 (50.0%)	15 (53.6%)	
**Coping Strategies**						0.72
Yes	50 (42.7%)	21 (42.9%)	13 (36.1%)	2 (50.0%)	14 (50.0%)	
No	67 (57.3%)	28 (57.1%)	23 (63.9%)	2 (50.0%)	14 (50.0%)	
**Lifestyle and Daily Routines**						0.73
Yes	61 (47.1%)	27 (55.1%)	16 (44.4%)	2 (50.0%)	16 (57.1%)	
No	56 (47.9%)	22 (44.9%)	20 (55.6%)	2 (50.0%)	12 (42.9%)	
**Social Skills Training Group**						0.83
Yes	46 (39.3%)	19 (38.8%)	16 (44.4%)	1 (25.0%)	10 (35.7%)	
No	71 (60.7%)	30 (61.2%)	20 (55.6%)	3 (75.0%)	18 (64.3%)	
**Behavioral Intervention**						0.95
Yes	44 (37.6%)	19 (38.8%)	14 (38.9%)	1 (25.0%)	10 (35.7%)	
No	73 (62.4%)	30 (61.2%)	22 (61.1%)	3 (75.0%)	18 (64.3%)	
**Rest and Sleep**						0.89
Yes	79 (67.5%)	33 (67.4%)	25 (69.4%)	2 (50.0%)	19 (67.9%)	
No	38 (32.5%)	16 (32.7%)	11 (30.6%)	2 (50.0%)	9 (32.1%)	
**Substance Use Group**						0.86
Yes	66 (56.4%)	28 (57.1%)	19 (52.8%)	3 (75.0%)	16 (57.1%)	
No	51 (43.6%)	21 (42.9%)	17 (47.2%)	1 (25.0%)	12 (42.9%)	
**Administration of Medications**						0.92
Yes	63 (53.9%)	26 (53.1%)	21 (58.3%)	2 (50.0%)	14 (50.0%)	
No	54 (46.1%)	23 (46.9%)	15 (41.7%)	2 (50.0%)	14 (50.0%)	
**Cognitive Retraining**						0.69
Yes	64 (54.7%)	26 (53.1%)	18 (50.0%)	3 (75.0%)	17 (60.7%)	
No	53 (45.3%)	23 (46.9%)	18 (50.0%)	1 (25.0%)	11 (39.3%)	
**Speech and Articulation Skills**						0.89
Yes	69 (59.0%)	30 (61.2%)	22 (61.1%)	2 (50.0%)	15 (53.6%)	
No	48 (41.0%)	19 (38.8%)	14 (38.9%)	2 (50.0%)	13 (46.4%)	
**Anxiety/Stress Management**						0.91
Yes	57 (48.7%)	24 (49.0%)	16 (44.4%)	2 (50.0%)	15 (53.6%)	
No	60 (51.3%)	25 (51.0%)	20 (55.6%)	2 (50.0%)	13 (46.4%)	
**Set-up of Medical Equipment**						0.97
Yes	61 (52.1%)	25 (51.0%)	20 (55.6%)	2 (50.0%)	14 (50.0%)	
No	56 (47.9%)	24 (49.0%)	16 (44.4%)	2 (50.0%)	14 (50.0%)	

* *p*-value obtained by chi-square test.

## Data Availability

Data can be obtained upon a reasonable request from the corresponding author.

## Supplementary Materials

The following supporting information can be downloaded at: https://www.mdpi.com/article/10.3390/ijerph21080974/s1, Supplementary File S1: Self-developed Questionnaire; Supplementary File S2: Occupational Therapy Referral Form.
