# Supplementary material for: Psychiatrists’ Insights on Integrating Occupational Therapy in Mental Health Care: A Multisite Middle Eastern Study
Source: Int J Environ Res Public Health. 2024 Jul 26;21(8):974. doi: 10.3390/ijerph21080974 (PMC11354036; doi:10.3390/ijerph21080974)
Supplement: Supplementary file 1 [file ijerph-21-00974-s001.zip › ijerph-3060390-supplementary.pdf]

Supplementary File S1: Self-developed Questionnaire

Part 1: Demographics

1- **Age……….**

2- **Gender:**
   - o Male
   - o Female

3- **Nationality:**
   - o Kuwaiti
   - o Egyptian
   - o Others (specify):……………………………………………………………………………………………….

4- **Educational level:**
   - o Bachelor
   - o Master
   - o Ph.D

5- **How many Years of clinical experience have You had:**
   - o Less than 1 year
   - o 1-5 years
   - o 5-10 years
   - o 11-15 years
   - o 16-20 years
   - o 21-25 years
   - o 26-30 years
   - o More than 30 years

6- **List the most common diagnoses seen in your practice (in order from most to least common)**

a. First most common:………………………………………………………………………………………….

b. Second most common:…………………………………………………………………………………….

c. Third most common:……………………………………………………………………………………….

d. Fourth most common:……………………………………………………………………………………..

e. Fifth most common:………………………………………………………………………………………..

7- Have you heard about occupational therapy?

- Yes
- No

8- How many years of experience have You had working with occupational therapists:

- No experience
- Less than one year
- 1-5 years
- 6-10 years
- More than 10 years

9- Have You attended any seminar/workshop related to occupational therapy:

- Yes
- No

10- If yes, how many times:

- Once
- Twice
- Three times
- More than three times

11- On average, how many patients do you refer to occupational therapy services per week:

- None
- 1-5
- 5-10
- > 10

<u>Part 2: Self-perception of occupational therapy autonomy:</u>

1- I know the **role and contributions** of occupational therapy in **<u>psychiatric rehabilitation:</u>**

- Strongly disagree
- Disagree
- Not sure
- Agree
- Strongly agree

2- I know the **<u>key difference</u>** between **Occupational Therapist** and **Psychologist**:

- Strongly disagree
- Disagree
- Not sure
- Agree
- Strongly agree

3- I know the **key difference** between **Occupational Therapist** and **social worker**:
- Strongly disagree
- Disagree
- Not sure
- Agree
- Strongly agree

Part 3: Knowledge about major components of occupational therapy intervention focus

Which of the followings are major components of occupational therapy intervention focus in mental health practice (Choose only the ones that apply)

- ☐ Activities of Daily Living
- ☐ Counseling
- ☐ Recreation/leisure
- ☐ Solving Family Problems
- ☐ Sensory Integration Group
- ☐ Psychological Assessments
- ☐ Thermotherapy
- ☐ Life Style and Daily Routines
- ☐ Behavioral Intervention
- ☐ Drug/Alcoholic group
- ☐ Cognitive retraining
- ☐ Anxiety/Stress Management
- ☐ Gait Training
- ☐ Academic Skills Training
- ☐ Physical Exercise
- ☐ Assertiveness training
- ☐ Helping patients to find jobs
- ☐ Pre/Vocational Training
- ☐ Coping Strategies
- ☐ Social skills training group
- ☐ Rest and Sleep
- ☐ Administration of Medications
- ☐ Speech and Articulation Skills
- ☐ set-up of medical equipment

Part 4: Attitudes toward occupational therapy

1- I believe occupational therapy is **an equally important health care profession as other health care professions** related to the rehabilitation of patients in mental health practice:

- Strongly disagree
- Disagree
- Not sure
- Agree
- Strongly agree

2- I **value the role of occupational therapy** in the rehabilitation of patients in mental health practice:

- Strongly disagree
- Disagree
- Not sure
- Agree
- Strongly agree

3- I would **refer** patients to **receive occupational therapy services in mental health practice**:

- Strongly disagree
- Disagree
- Not sure
- Agree
- Strongly agree

Part 5: Self-efficacy regarding occupational therapy

1- I am **motivated** to work with occupational therapists as part of the **rehabilitation team**:

- Strongly disagree
- Disagree
- Neutral
- Agree
- Strongly agree

2- I am **confident** in the skills and contributions of occupational therapist for evaluating and treating patients with mental illness:

- Strongly disagree
- Disagree
- Neutral
- Agree
- Strongly agree

Part 6: Satisfaction with your knowledge base of occupational therapy contributions in patients' rehabilitation

1- I am **satisfied** with my **knowledge base regarding occupational therapy contributions in mental health practice:**

- Strongly disagree
- Disagree
- Neutral
- Agree
- Strongly agree

Thank you for your time to fill out the survey

Supplementary File S2: Occupational Therapy Referral Form

Occupational Therapy Referral Form

HOSPITAL: ______________________ Hosp. No. ☐☐ ☐☐ ☐☐

Patient's Name: .... File No.: ....

D.O.B: .... Gender: Male / Female

Nationality: .... Guardian: ....

Phone Number: .... / ....

Diagnosis: ....

☐ IN-PATIENT ☐ OUT-PATIENT

Brief History:..

....

....

REASON FOR REFERRAL TO OCCUPATIONAL THERAPY:

- ☐ Activities of Daily Living
- ☐ Life Style and Daily Routines
- ☐ Recreation/leisure
- ☐ Drug/Alcoholic group
- ☐ Sensory Integration Group
- ☐ Cognitive retraining
- ☐ Anxiety/Stress Management
- ☐ Academic skills Training
- ☐ Social skills training group
- ☐ Anger management group
- ☐ Assertiveness training
- ☐ Behavior Intervention
- ☐ Pre/Vocational Training
- ☐ Oncology/Palliative care

Other (specify): ....

PRECAUTIONS (suicidal tendencies, aggressive behavior, etc.):

....

ANY OTHER RELEVANT INFORMATION: ....

REFERRING PSYCHIATRIST: ....

PSYCHIATRIST'S SIGNATURE & STAMP:

.... **DATE:** / /
